# Nystose regulates the response of rice roots to cold stress via multiple signaling pathways: A comparative proteomics analysis

**DOI:** 10.1371/journal.pone.0238381

**Published:** 2020-09-03

**Authors:** Zijie Zhang, Wenfei Xiao, Jieren Qiu, Ya Xin, Qinpo Liu, Huizhe Chen, Yaping Fu, Huasheng Ma, Wenyue Chen, Yuqin Huang, Songlin Ruan, Jianli Yan

**Affiliations:** 1 Institute of Crop Science, Hangzhou Academy of Agricultural Sciences, Hangzhou, China; 2 College of Agriculture and Food Science, Zhejiang Agriculture & Forestry University, Hangzhou, China; 3 National Key Laboratory of Rice Biology, China National Rice Research Institute, Chinese Academy of Agricultural Sciences, Hangzhou, China; 4 College of Agriculture and Biotechnology, Zhejiang University, Hangzhou, China; University of Western Sydney, AUSTRALIA

## Abstract

Small fructans improve plant tolerance for cold stress. However, the underlying molecular mechanisms are poorly understood. Here, we have demonstrated that the small fructan tetrasaccharide nystose improves the cold stress tolerance of primary rice roots. Roots developed from seeds soaked in nystose showed lower browning rate, higher root activity, and faster growth compared to seeds soaked in water under chilling stress. Comparative proteomics analysis of nystose-treated and control roots identified a total of 497 differentially expressed proteins. GO classification and KEGG pathway analysis documented that some of the upregulated differentially expressed proteins were implicated in the regulation of serine/threonine protein phosphatase activity, abscisic acid-activated signaling, removal of superoxide radicals, and the response to oxidative stress and defense responses. Western blot analysis indicated that nystose promotes the growth of primary rice roots by increasing the level of RSOsPR10, and the cold stress-induced change in RSOsPR10levelis regulated by jasmonate, salicylic acid, and abscisic acid signaling pathways in rice roots. Furthermore, OsMKK4-dependentmitogen-activated protein kinase signaling cascades may be involved in the nystose-induced cold tolerance of primary rice roots. Together, these results indicate that nystose acts as an immunostimulator of the response to cold stress by multiple signaling pathways.

## Introduction

Fructans are acid-labile, water-soluble, polydisperse fructose polymers with a sucrose starter unit. They represent the major reserve carbohydrates in approximately 15% of flowering plants [1]. Fructans can be linear or branched, and their degree of polymerization (DP) ranges from 3 to 300, depending on the species and developmental stage of the plant as well as environmental conditions [2]. Fructans are classified according to their glycosidic linkages: β(2,1), β(2,6), or both. There are two forms of trisaccharide fructans, 1-kestotriose, 6-kestotriose, and 6G-kestotriose (neokestose); they belong to inulin- and levan-type fructans, respectively. Tetrasaccharide nystose is a fructooligosaccharide with two fructose molecules linked via β(2,1) bonds to the fructosyl moiety of sucrose. Nystose typically occurs in the eudicot family Asteraceae and the monocot families Asparagaceae, Liliaceae, and Poaceae; however, it is absent in rice [3].

It has been suggested that fructans function as reserve carbohydrates deposited in the underground storage organs of dicots and above-ground parts of monocots [2, 4, 5]. Additionally, fructans regulate flower opening. Closed petals of *Campanula rapunculoides* and *Hemerocallis* have a high fructan content, while no fructan is present in the petals of opened flowers [6, 7]. Fructanexohydrolases quickly release a significant amount of fructose, facilitating water inflow and flower opening by lowering the osmotic pressure. In addition, fructans protect plants from cold stress and water deficit [8–11] and maintain the sucrose concentration necessary for photosynthesis and transport [12]. These properties of fructans led to the hypothesis that they act as membrane stabilizers. Indeed, by interacting with membrane lipids, fructans increase membrane stability during freezing and cellular dehydration [10, 13–17]. Fructans also scavenge hydroxyl radicals (·OH) generated by the activity of tonoplast-associated class III peroxidase [18, 19]. In contrast to glucose-, fructose-, and sucrose-specific signaling pathways, which are well characterized in plants [20–22], the cold stress-related fructan signaling pathways are poorly understood.

Cold stress, including chilling (0–15°C) and freezing (< 0°C), is a major environmental factor affecting plant growth and development, and influencing crop yield [23–27]. Cold stress triggers numerous physiological responses in plants, including membrane rigidification, suppression of nutrient absorption, reduced photosynthetic rate, growth inhibition [28], transient increases in the levels of hormones such as abscisic acid (ABA) and jasmonic acid (JA) [29], changes in membrane lipid composition [30], accumulation of compatible osmolytes such as soluble sugars, betaine, and proline [31–33], and increased formation of antioxidants [34]. Cold stress signaling pathways involve calcium signaling and mitogen-activated protein kinase (MAPK) cascade pathways, hormone-mediated signaling (e.g., JA, ABA), and reactive oxygen species (ROS) signaling [24].

The development of molecular technologies and proteomics enables a comprehensive analysis of changes in plant protein expression in response to cold stress. Kawamura and Uemura (2003) were the first to apply mass spectrometry to elucidate the relationship between plasma membrane proteins and cold acclimation. They successfully identified 38 relevant proteins in *Arabidopsis* [35], including early response to dehydration proteins ERD10 and ERD14, members of the dehydrins family that protect against cold-induced dehydration [36, 37]. In rice seedlings exposed to the stress of a progressively decreasing temperature, from normal t to 15, 10, and 5°C, 41 cold-responsive proteins were identified using 2-D electrophoresis combined with MALDI-TOF MS or ESI/MS/MS. These proteins were involved in protein quality control mediated by chaperones and proteases, and in the enhancement of cell wall components [38]. Neilson and coworkers identified 236 cold-responsive proteins compared using label-free quantification to 85 using iTRAQ, with only 24 common proteins identified. Functional analysis revealed cold stress-related differential expression of proteins involved in transport, photosynthesis, generation of precursor metabolites, histones, and vitamin B biosynthesis [39]. These findings can guide molecular breeding strategies aiming at the improvement of tolerance to cold in crops [40].

Microarray technology provides a powerful tool for genome-wide identification of genes involved in the response of rice to cold stress and dissecting the mechanisms responsible for their effects. Comparative transcriptome analysis of two rice genotypes with contrasting responses to cold stress revealed that the major effect of low temperature was the upregulation of gene expression in the cold-tolerant genotype, and downregulation in the cold-sensitive genotype [41]. Genome-wide profiling of gene expression in a cold-tolerant *japonica* variety identified 5557 differentially expressed genes (DEGs) at four distinct time points during moderate cold stress (8°C). Further analysis revealed that the glutathione system and the ABA signaling pathway played a dominant role in the cold stress response [42]. In the present study, integrated analysis of morphology, physiology, and comparative proteomics demonstrated that nystose regulates the response of rice roots to cold stress via multiple signaling pathways, including jasmonate, SA and ABA signaling pathways, and MAPK signaling cascades. These findings provide novel insights into the role of nystose-specific signaling pathways in the response of rice roots to cold stress.

## Materials and methods

### Plant materials and growth conditions

#### Rice growth conditions

Seeds of rice variety ‘Zhongzheyou 1’ were supplied by the WuWangNong Seed Group (Hangzhou, Zhejiang province, China). The seeds were soaked in 50 ml of water containing 75 mg/L nystose (Seebio Biotechnology Co., Ltd., Shanghai, China) for 1 d at 30°C, and rinsed with distilled water three times. Subsequently, 20 rice seeds were transferred to 50 ml of agar containing 75 mg/L nystose and incubated at 15°C for 7 d.

#### Rice cold treatment and recovery protocol

Rice seeds were soaked in 50ml water containing 75 mg/L nystose, 2.5 mg/L ABA, 2.5 mg/L JA, and 10 mg/L SA, respectively. After germinating for 40h at 30°C, 50 rice seedlings were transferred to germination boxes containing two layers of filter papers moistened with distilled water and incubated at 4°C for 24h and 48h. For the recovery, seedlings subjected to cold stress for 48 h were transferred to normal culture conditions.

### Measurement of primary root length

The length of primary roots in 20 control and treated plants was measured after 7 d of growth at 15°C.

### Determination of browning

The number of rice roots that turned from white to brown after 5d of recovering in normal culture conditions was counted. The percentage of browning was calculated as the number of browned roots/50*100%.

### TTC reduction assay

For the TTC assay, 50 root samples were cut into 1 cm segments and placed in 10 ml of TTC solution (5 ml 0.4% TTC and 5 ml 0.05 M sodium phosphate buffer, pH 7.4) for 2 h in the dark at 37°C. TTC solution was then drained, and the specimens were washed in sterile water. TTC was extracted in 10 ml of methanol for 1 d at 37°C, and its absorbance was measured at 485 nm. The amount of reduced TTC was calculated according to the standard curve prepared using 20, 40, 80, 120, 160, and 200 μg of TTC.

### Protein preparation

Rice roots (1 g) were ground in liquid nitrogen, suspended in 5 ml acetone with 10% (w/v) trichloroacetic acid and 0.07% (w/v) β-mercaptoethanol at -20°C for 1 h, and centrifuged for 30 min at 14,000 g. Pellets were resuspended in 0.07% (w/v) β-mercaptoethanol in acetone, incubated at -20°C for 1 h, and centrifuged14,000 g for 15 min at 4°C. This step was repeated three times. The pellets containing crude protein were dried and solubilized in lysis buffer (8 M urea, 100 mMTris-HCl (pH 8.5), and 1 mM PMSF) for 1 h at room temperature. The samples were then centrifuged15 min at 14,000g, and the supernatants were collected in 1.5 ml tubes. A 4 μl aliquot was used to measure protein concentration by the Bradford assay, using bovine serum albumin as the standard.

### Protein digestion

Protein samples (100 μg) were mixed with 50 mM NH_4_HCO_3_ to reach the final volume of 150 μl. Subsequently, 100mM stock solution of DTT was added for the final concentration of 10 mM, mixed at 600 rpm for 1 min, and incubated at 37°C for 1h. IAA (500 mM) was added to obtain the final concentration of 50 mM, mixed at 600 rpm for 1 min, and stored at room temperature in the dark for 30 min. The samples were then passed through a 10 kDa filter and centrifuged at 14,000 g for 15 min at 4°C. Next, 100 μl of trypsin buffer (2 μg trypsin in 100 μl NH_4_HCO_3_ buffer) was added, and the tryptic digestion was performed for 16–18 h at 37°C. The products of digestion were collected after centrifugation at 14,000g for 10 min and subsequent addition of 100 μl NH_4_HCO_3,_followed by centrifugation at 14,000g for 10 min. Peptides were desalted using the C18 tip (Thermo Fisher Scientific, Waltham, MA, USA).

### LC-MS/MS analysis

RP-HPLC separation was performed using a nanoflow HPLC instrument (ProxeonBiosystems, now Thermo Fisher Scientific) equipped with a self-packed tip column (75 μm × 150 mm; C18, 3.0 μm). A 120 min gradient was applied at a flow rate of 300 nl/min. For mass spectrometry, a Q-Exactive mass spectrometer (Thermo Fisher Scientific) equipped with a nanoelectrospray ion source (ProxeonBiosystems, now Thermo Fisher Scientific) was used. Data were acquired in the data-dependent “top10” mode, in which the ten precursor ions of the highest abundance were selected with at a high resolution (70,000 at m/z 200) from the full scan (300–2000 m/z) for HCD fragmentation. Precursor ions with single or unassigned charges information were excluded. The resolution for the MS/MS spectra was set to 17500 at m/z 200, with a target value of 2E5 at enabled AGC control. The isolation window was set to 2.0 m/z, with a lock mass option enabled for the 445.120025 ion. The normalized collision energy was 29%.

### Database search and bioinformatics

MS data were analyzed using MaxQuant software version 1.6.0.1 to obtain matched protein datasets for label-free quantification (LFQ) (S4 File). The data were searched against the *Oryza sativa* subsp. *japonica* database (122,587 total entries). The main search parameters were as follows: main search ppm: 6, missed cleavage: 2, MS/MS tolerance ppm: 20, enzyme: trypsin, variable modification: oxidation (M) and acetyl (protein N-term), decoy database pattern: reverse, LFQ: true, LFQ min. ratio count: 1, match between runs: 2 min, peptide FDR: 0.01, and protein FDR: 0.01 [43]. DEP analysis was performed using the Perseus software (version 1.6.5.0). KEGG and GO analyses of the differentially expressed proteins (DEPs)were performed in DAVID 6.8 (https://david.ncifcrf.gov/). Figures were constructed in the R environment using the ggplot2 package.

### Preparation of antibodies and Western blot analysis

Peptide fragments were examined as protein-surface antigens (for the sequences, see S5 File). Peptides containing an additional N-terminal cysteine were synthesized, purified on resin, and used to raise polyclonal antibodies in rabbits (HuaAn Biotechnology Co., Ltd., Hangzhou, China, or Beijing Protein Innovation Co.,Ltd., Beijing, China). For Western blotting, rice roots (0.5 g) were ground into a fine powder in liquid nitrogen and suspended in 2 ml of protein extraction buffer containing 50 mM Tris-HCl, pH8.0, 1 mM EDTA, 10 mM NaCl, 1% SDS, 0.5% (v/v) 2-mercaptoethanol, 0.1 mM PMSF, 0.1 mM DTT and 0.1% (v/v) Triton X-100. The suspension was ground until the powder was fully homogenized. Mixtures were then centrifuged at 14,000 g for 15 min at 4°C, and supernatants were transferred into 5 ml centrifuge tubes. Protein concentration was determined using the *RC DC* protein assay kit II (Bio-Rad, Hercules, CA, USA). Samples containing 20 μg of total protein were subjected to electrophoresis on 15% SDS-polyacrylamide gel. Separated proteins were transferred to PVDF membranes, which were blocked with 5% (w/v) skimmed milk. Blots were incubated with rabbit antibodies diluted 1:1000 in TBST (25 mM Tris base pH 8.0, 140 mM NaCl, 3 mM KCl, 0.05% (v/v) Tween 20) for 1 h and washed three times for 5 min in TBST. Subsequently, the blots were probed with HRP-labeled goat anti-rabbit IgG (H+L) diluted 1:5000. Reactive bands were visualized using ECL (Multiscience Biotech Co., Ltd., Hangzhou, China).

### Statistical analysis

Each treatment was performed in three independent experimental replicates, and the data are presented as the mean ± SE. The analysis of variance (ANOVA) was performed using Duncan’s multiple range test. Percentages were transformed according to y = arcsin [SQR(x/100)]. All data were analyzed according to a factorial model and replicates as random effects. The treatments were compared using the least significant difference at the 0.05 confidence level.

## Results

### Effect of nystose on the phenotypes and physiological parameters of rice primary roots under normal conditions and cold stress

**Fig 1:**
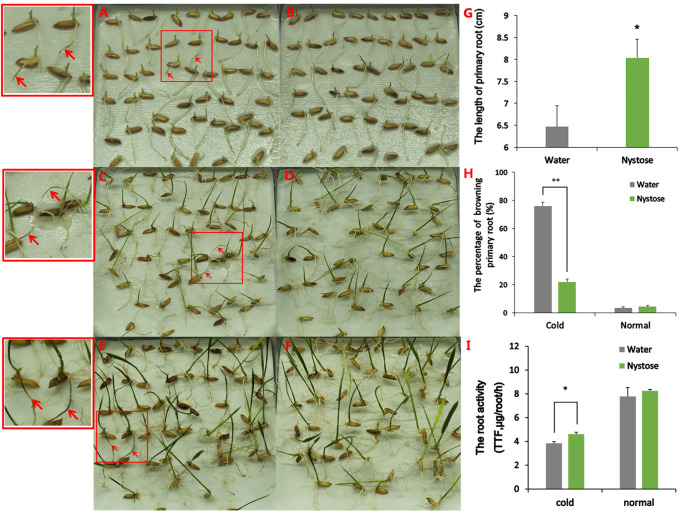
Phenotypes and physiological indices of rice roots under normal growth and cold stress. (A, C, E) Phenotypes of primary rice roots from seeds soaked in water, grown at 4°C for 2 d, and allowed to recover at 25°C for 1 d (A), 3 d (C), and 5 d (E). Red arrows in the boxes point to the brown discoloration of primary rice roots in the control group. Selected areas indicated by small red boxes are shown at higher magnification large red boxes. (B, D, F). Phenotypes of primary rice roots from seeds soaked in 75 mg/L nystose, grown at 4°C for 2 d, and allowed to recover at 25°C for 1 d (B), 3 d (D) and 5 d (F). (G) Length of primary rice roots after growing at 15°C for 10 days after sowing. Twenty independent experimental replicates were analyzed for each treatment, and data are presented as the mean ± SE. An independent t-test was performed to determine the differences between the SSN and SSW groups (*P<0.05). (H) The fraction of browning primary roots. Each experiment was performed in triplicate, and data are presented as the mean ± SE. An independent t-test was performed to determine the differences between groups (**P<0.01). (I) Metabolic activity of the roots. Each experiment was performed in triplicate, and data are indicated as the mean ± SE. An independent t-test was performed to determine the difference between groups (*P<0.05).

Rice seeds were soaked in nystose solution (SSN, treatment group) or water (SSW, control group) at 4°C for 2 d and allowed to recover at 25°C for 1 d, 3 d, and 5 d. After 1 day of the recovery, the apical parts of the primary roots in the SSW group showed browning (Fig 1A), and the degree of root tip browning gradually increased with recovery time (Fig 1C and 1E). The majority of primary root tips in the control group exhibited a typical white phenotype (Fig 1B, 1D and 1F). Under normal growth conditions, no significant differences in the phenotypes of primary root tips were observed between the two groups, and the tips had a normal white phenotype (S1 Fig). Thus, the percentage of browning primary roots in the SSW group was higher than in the SSN group (Fig 1H). The ability of roots to reduce TTC was then used as an indicator of their tolerance to cold. The results of TTC staining assays documented the metabolic activity of primary roots in the treatment group was higher in the control group under cold stress, while no significant differences were observed between SSN and SSW (Fig 1I). In addition, the length of the primary roots in the SSN group was greater than in the control group after growing at 15°C for 10 days after sowing (Fig 1G). Together, these data suggest that nystose improves the cold tolerance of primary roots of rice.

### Protein expression profiles of nystose-treated and control primary roots under normal growth conditions and cold stress

**Fig 2:**
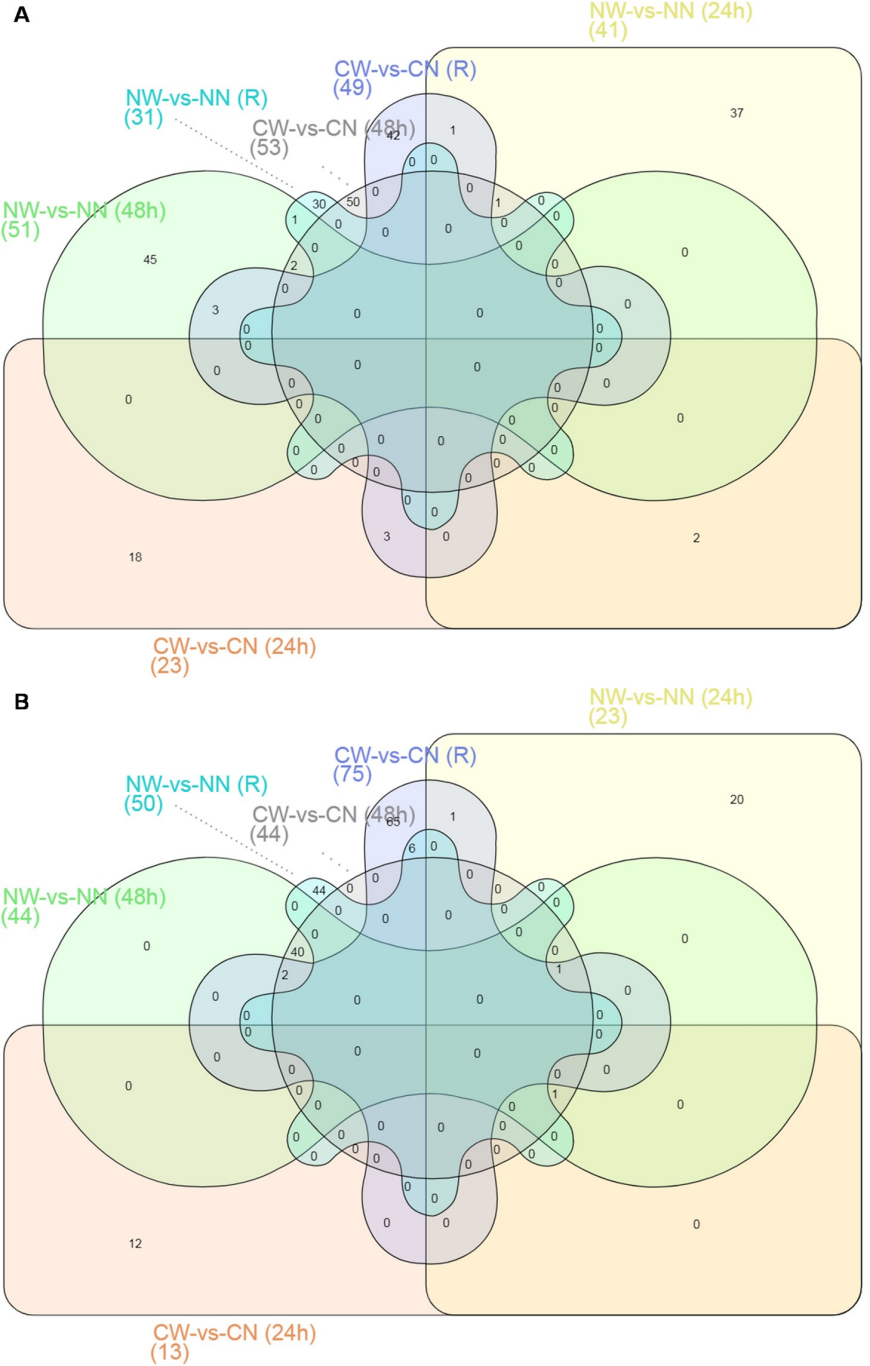
Protein expression profiles in control and nystose-treated rice roots under normal growth conditions and cold stress. A: Venn diagram of the number of DEPs with fold change >1.5 (P<0.05). B: Venn diagram of the number of DEPs with fold change<2/3 (P<0.05). NW-vs-NN (24 h): rice roots that grew at 25°C for 24 h after treatment with water and nystose. CW-vs-CN (24 h): rice roots that grew at 4°C for 24 h after treatment with water and nystose. NW-vs-NN (48h): rice roots that grew at 25°C for 48 h after treatment with water and nystose. CW-vs-CN (48 h): rice roots that grew at 4°C for 48 h after treatment with water and nystose. NW-vs-NN (R): rice roots that grew at 25°C for 7 d after treatment with water and nystose. CW-vs-CN (R): rice roots that grew at 4°C for 2 d and then at 25°C for 5 d after treatment with water and nystose.

To determine the molecular mechanisms underlying the ability of nystose to improve the cold tolerance of primary roots of rice, comparative proteomics analysis was performed. The objective was to identify DEPs in the primary roots in the treatment and control groups under normal growth conditions (25°C for 24 h, 48 h, and 7 d), cold stress (4°C for 24 h and 48 h) and recovery from cold stress (25°C for 5d after growth at 4°C for 48 h). As shown in S2 Fig, a total of 916 DEPs were identified, with similar numbers of upregulated and downregulated proteins. Among them, 497 DEPs, including 248 upregulated DEPs and 249 downregulated DEPs, were characterized by fold-change ≥ 1.5 (Fig 2). In addition, the number of upregulated proteins was greater than the number of downregulated proteins under normal growth conditions and cold stress, while the number of upregulated proteins was lower than the number of downregulated proteins under normal growth conditions and recovery from cold stress (Fig 2, S2 Fig).

### GO classification of differentially expressed proteins

To explore the biological functions of the 497 identified DEPs, GO classification was conducted. The DEPs were assigned to 18 cell components, classified into 22 functional categories, and involved in 27 biological processes (S3 Fig). Specifically, the 248 upregulated DEPs were assigned to 13 cell components, classified into 14 functional categories, and involved in 18 biological processes (S5 Fig), while the 249 downregulated DEPs were assigned to 10 cell components, classified into 18 functional categories, and involved in 16 biological processes (S4 Fig). Some DEPs were classified into 6 functional categories, which may be closely related to stress responses. These categories included abscisic acid binding, receptor activity, protein phosphatase inhibitor activity, FK506 binding, signal recognition particle binding, and 12-oxophytodienoate reductase activity. The DEPs were also involved in the regulation of protein serine/threonine phosphatase activity, chaperone-mediated protein folding, and oxylipin biosynthesis (S3A and S3F Fig). Some of the upregulated DEPs were within functional categories of abscisic acid binding, receptor activity, protein phosphatase inhibitor activity, superoxide dismutase activity, peroxidase activity, and kinase activity. Upregulated DEPS were also implicated in the regulation of serine/threonine protein phosphatase activity, abscisic acid-activated signaling, removal of superoxide radicals, response to oxidative stress, and defense responses to bacteria (S5A, S5C and S5D Fig). The downregulated DEPs belonged to 3 functional categories: glutathione transferase activity, signal recognition particle binding, and 12-oxophytodienoate reductase activity. Additionally, downregulated DEPs were involved in glutathione metabolism, type I hypersensitivity, and defense responses to fungi (S6A and S6F Fig). It is generally accepted that root function is closely related to dehydrogenase activity [44, 45]. In this regard, we have identified DEPs with dehydrogenase activity (S3E and S3F Fig), which are likely to be responsible for the higher activity of the root after the treatment with nystose.

### Pathway analysis of the differentially expressed proteins

To identify potential target genes of the 497 DEPs detected in primary roots, pathway analysis was performed for all 248 upregulated and 249 downregulated proteins. The 497 DEPs are involved in 16 distinct metabolic pathways: glutathione metabolism, ribosome, carbon metabolism, valine, leucine, and isoleucine degradation, citrate cycle (TCA cycle), synthesis of antibiotics, pyruvate metabolism, RNA transport, glycolysis/gluconeogenesis, galactose metabolism, starch and sucrose metabolism, pentose and glucuronate interconversions, ascorbate and aldarate metabolism, synthesis of amino acids, metabolic pathways, and the biosynthesis of secondary metabolites (S4 Fig). Upregulated and downregulated DEPs were involved in 11 and 14 distinct metabolic pathways (S7 & S8 Figs), respectively. Of these pathways, 7 were shared by up- and downregulated DEPs: synthesis of antibiotics, pyruvate metabolism, carbon metabolism, glycolysis/gluconeogenesis, ribosome, carbon fixation in photosynthetic organisms, the biosynthesis of secondary metabolites. Importantly, the glutathione and galactose metabolism associated with upregulated DEPs (S7A and S7C Fig) and the ascorbate, aldarate, and alpha-linolenic acid metabolism associated with downregulated DEPs (S8E Fig) is closely related to stress responses.

### Validation of differentially expressed candidate proteins

**Fig 3:**
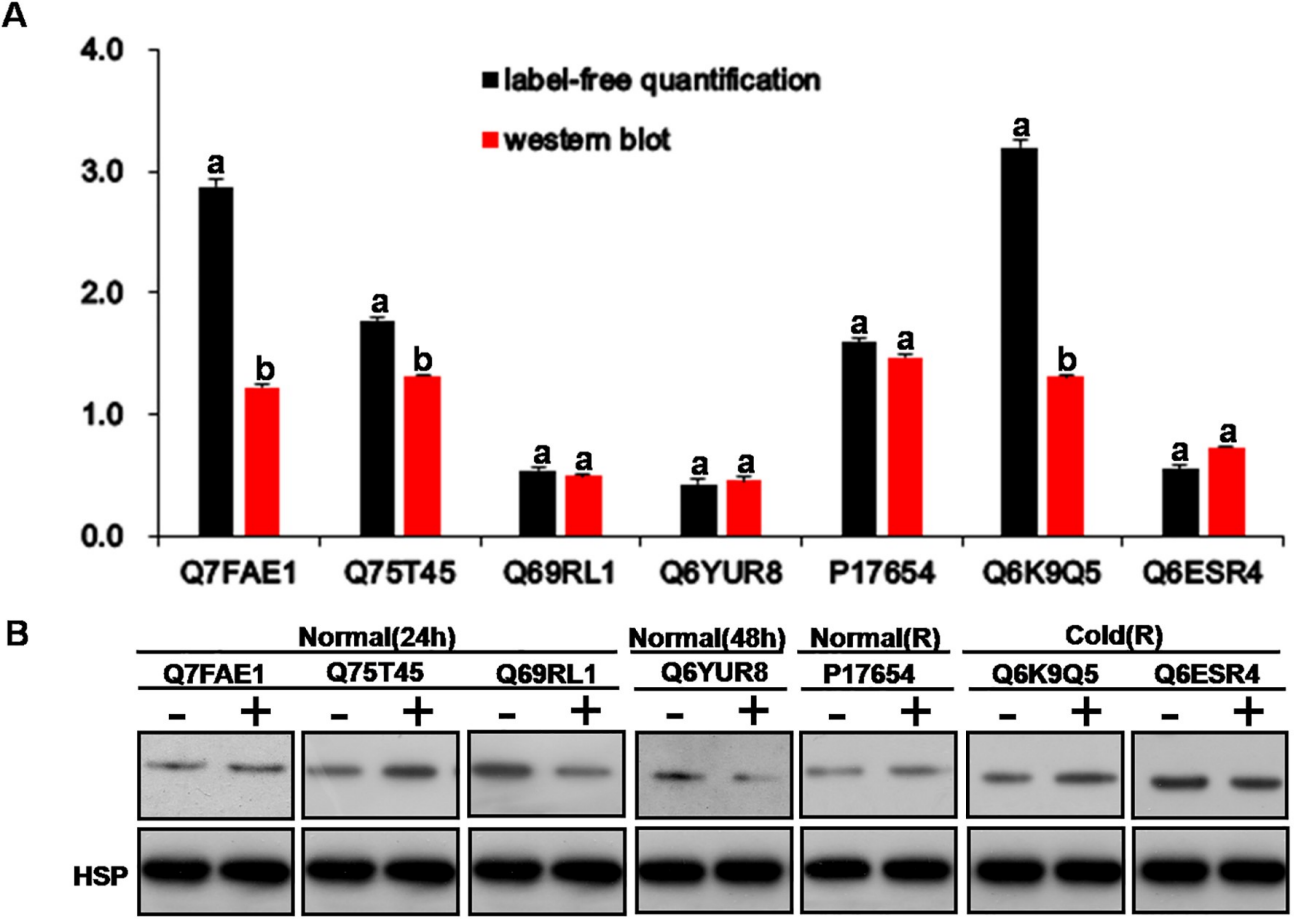
Validation of DEPs. (A) Comparison of fold-change of DEPs obtained by label-free quantification and Western blot analysis. The values on the y-axis indicate fold-change. (B) Western blot analysis of DEPs showing four upregulated proteins, including Q7FAE1 (momilactone A synthase), Q75T45 (RSOsPR10),P17654 (alpha-amylase) and Q6K9Q5 (serine/threonine-protein phosphatase), and three downregulated proteins, including Q69RL1 (GST), Q6YUR8 (CSP1) and Q6ESR4 (DHN1). Four DEPs, including Q7FAE1, Q75T45, Q69RL1, and Q6YUR8, of rice roots grown at 25°C for 24 h and 48 h after the treatment with water and nystose. Three DEPs, including P17654, Q6K9Q5, and Q6ESR4, of rice roots grown at 25°C for 7 d and 4°C for 2 d and then at 25°C for 5 d after the treatment with water and nystose. Normal (24 h) represents rice roots grown at 25°C for 24 h after the treatment with water and nystose. Normal (48 h) represents rice roots grown at 25°C for 24 h after the treatment with water and nystose. Normal (R) represents rice roots grown at 25°C for 7 d after the treatment with water and nystose. Cold (R) represents rice roots grown at 4°C for 2 d and then at 25°C for 5 d after the treatment with water and nystose. “+” indicates soaking of the seeds in 75 mg/L nystose, “-” indicates soaking of the seeds in water.

To validate the proteome datasets and the patterns of the DEPs revealed by label-free quantification (S1–S3 Files), Western blot analysis was performed to document the changes in expression of 7 DEPs, including 4 upregulated and 3 downregulated DEPs (Fig 3, S2 & S3 Files). This experiment demonstrated that 4 proteins in the roots of the nystose-treated group, Q7FAE1 (momilactone A synthase), Q75T45 (RSOsPR10), P17654 (alpha-amylase), and Q6K9Q5 (serine/threonine-protein phosphatase), had a higher level of expression than in the untreated control group after 24 h and 48 h of cold stress and growth recovery, respectively (Fig 3A and 3B). Three DEPs downregulated in the roots of the nystose-treated group, Q69RL1 (GST), Q6YUR8 (CSP1), and Q6ESR4 (DHN1), had a lower level of expression than in the untreated control group at 24 h of cold stress (Fig 3B). These results are consistent with the quantification data of label-free proteomics in primary rice roots from both groups.

## Discussion

Rice is a cold-sensitive cereal crop. When exposed to low temperatures, rice plants suffer from chilling injury, which leads to growth inhibition and the browning of leaves and roots, decreasing grain yield and quality [46–48]. The present study identified the browning phenotype in primary roots of untreated rice grown at 4°C for 2 d and allowed to recover at 25°C for 1 d, 3 d, and 5 d. The browning indicated chilling injury, growth inhibition, and decreased root activity. The majority of primary root tips in the nystose-treated group exhibited normal white phenotype and higher activity, indicating that nystose protects against chilling injury. Furthermore, a primary root-based stress tolerance-induced system was developed. The system has the advantages of simple operation, clear phenotypic identification, and the lack of interference from housekeeping proteins in isolation and identification, providing an effective tool for future proteome-level research on the mechanisms underlying the impact of nystose on the regulation of the responses of rice roots to cold stress.

The possibility has been raised that fructans act as important immunostimulators [49, 50]. In the current investigation, 4 antioxidant enzymes, including one Cu-Zn superoxide dismutase (P93407) and three peroxidases (A0A0P0X9R7,Q5U1P7, Q5U1Q2) showed higher levels in nystose-treated primary roots grown at 25°C and 4°C for 48h than in untreated plants (S1 File). It is generally accepted that these antioxidant enzymes are directly involved in the scavenging of excess free radicals in primary rice roots, and may contribute to their protection against browning under cold stress. Fructans enhance membrane stability at low temperatures by interacting with membrane lipids [10, 13–17] and scavenging hydroxyl radicals (·OH) generated by the activity of tonoplast-associated class III peroxidase [18, 19]. Therefore, nystose is likely to participate in antioxidant mechanisms in primary rice roots, protecting them from browning under cold stress.

**Fig 4:**
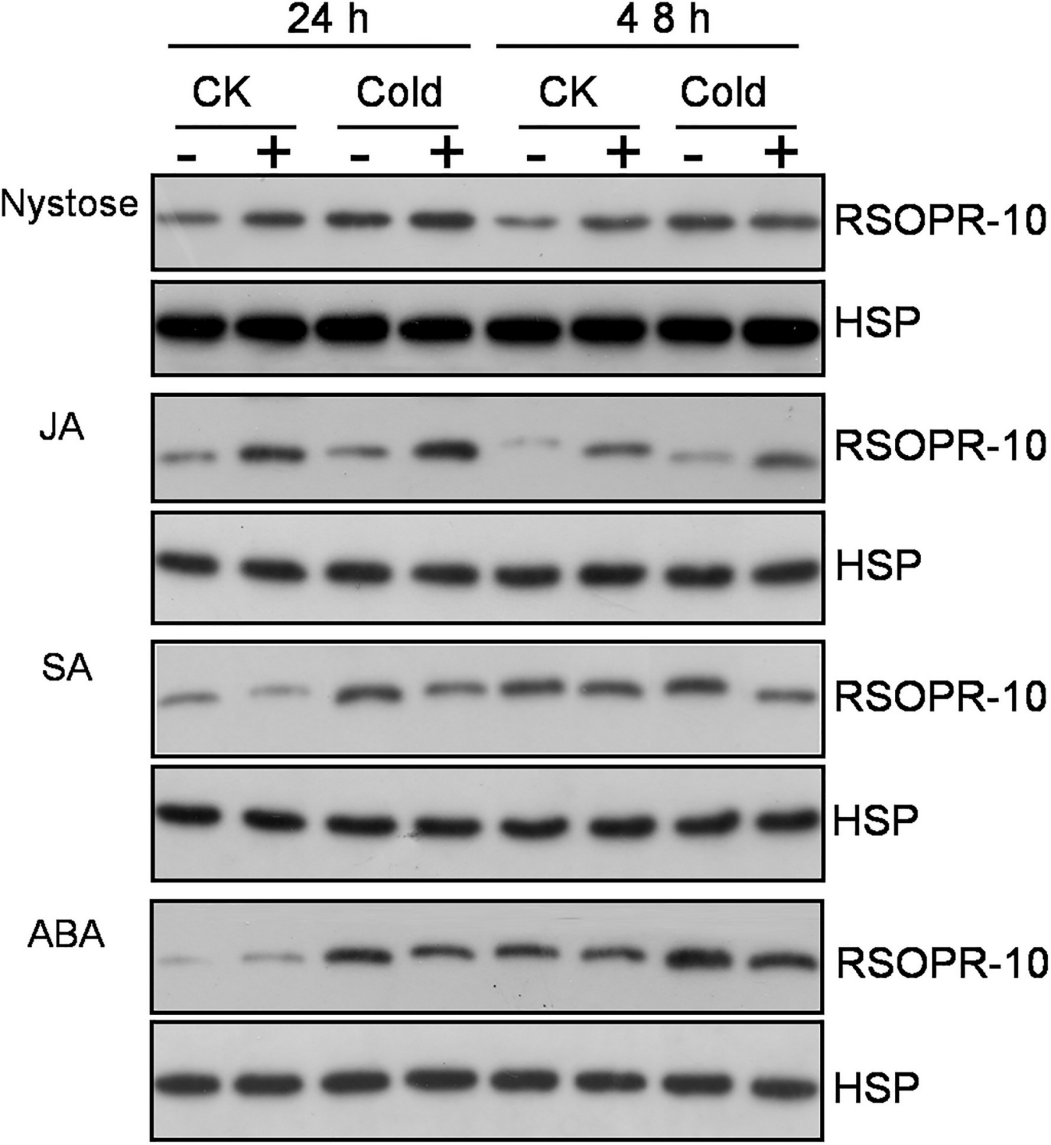
Western blot analysis of RSOsPR10 expression in response to nystose, JA, SA, and ABA. CK: rice roots grown at 25°C for 2 d after the treatment. Cold: rice roots grown at 4°C for 2 d after the treatment. “-“: seeds treated with water; “+”: seeds treated with 2.5 mg/L JA, 5 mg/L SA, 2.5 mg/L ABA, or 75mg/L nystose.

Interestingly, we have identified 5 upregulated DEPs, including Q10MP7 (PR10c), Q2QNS7 (PR10a), Q75T45 (RSOsPR10), Q941F5 (PR4), and Q84J76 (putative pathogenesis-related protein) (S1 File). These DEPs are likely to be involved in immune and stress responses. The expression of RSOsPR10, a root-specific pathogenesis-related (PR) protein, is rapidly induced in roots by salt, drought stress, and blast fungus infection, possibly by the activation of the JA signaling [51]. Also, RSOsPR10 is strongly induced by JA and the ethylene (ET) precursor 1-aminocyclopropane-1-carboxylic acid (ACC), while salicylic acid (SA) almost completely suppresses these effects [52]. Similarly, OsERF1, a transcription factor in the JA/ET pathway, is regulated by JA, ACC, and SA [50], but is induced before RSOsPR10 upon the treatment with JA and ACC [52]. Therefore, it was proposed that the environmental stress-induced changes in RSOsPR10 expression in rice roots are antagonistically regulated by JA/ET and SA [52]. The present work documented that under normal conditions or cold stress, the expression of RSOsPR10 was strongly upregulated by nystose and JA, but was significantly inhibited by SA (Fig 4). Under normal conditions, no significant differences in RSOsPR10 expression were observed between rice roots treated with water or ABA, while under cold stress, RSOsPR10 level was significantly lower in ABA-treated roots than in the controls (Fig 4). Indeed, the integration of our datasets was in agreement with previous findings that fructans act as essential immunostimulators involved in universal antioxidant mechanisms in plants. Recently, RSOsPR10 was shown to mediate environmental stress tolerance by increasing root growth and development [53]. Here, we have demonstrated that nystose increases RSOsPR10 level and promotes the growth of primary rice roots under cold stress. Together, these data suggest that nystose improves the tolerance of primary rice roots to low temperatures by upregulating the expression of RSOsPR10. In addition, RSOsPR10 expression is induced by cold stress, contrasting the previous report that *RSOsPR10*mRNA does not accumulate after the exposure to low temperature [51]. However, inconsistencies in the correlation between gene transcription and protein translation can occur, and that report [51] did not evaluate the expression pattern of RSOsPR10 protein under cold stress.

**Fig 5:**
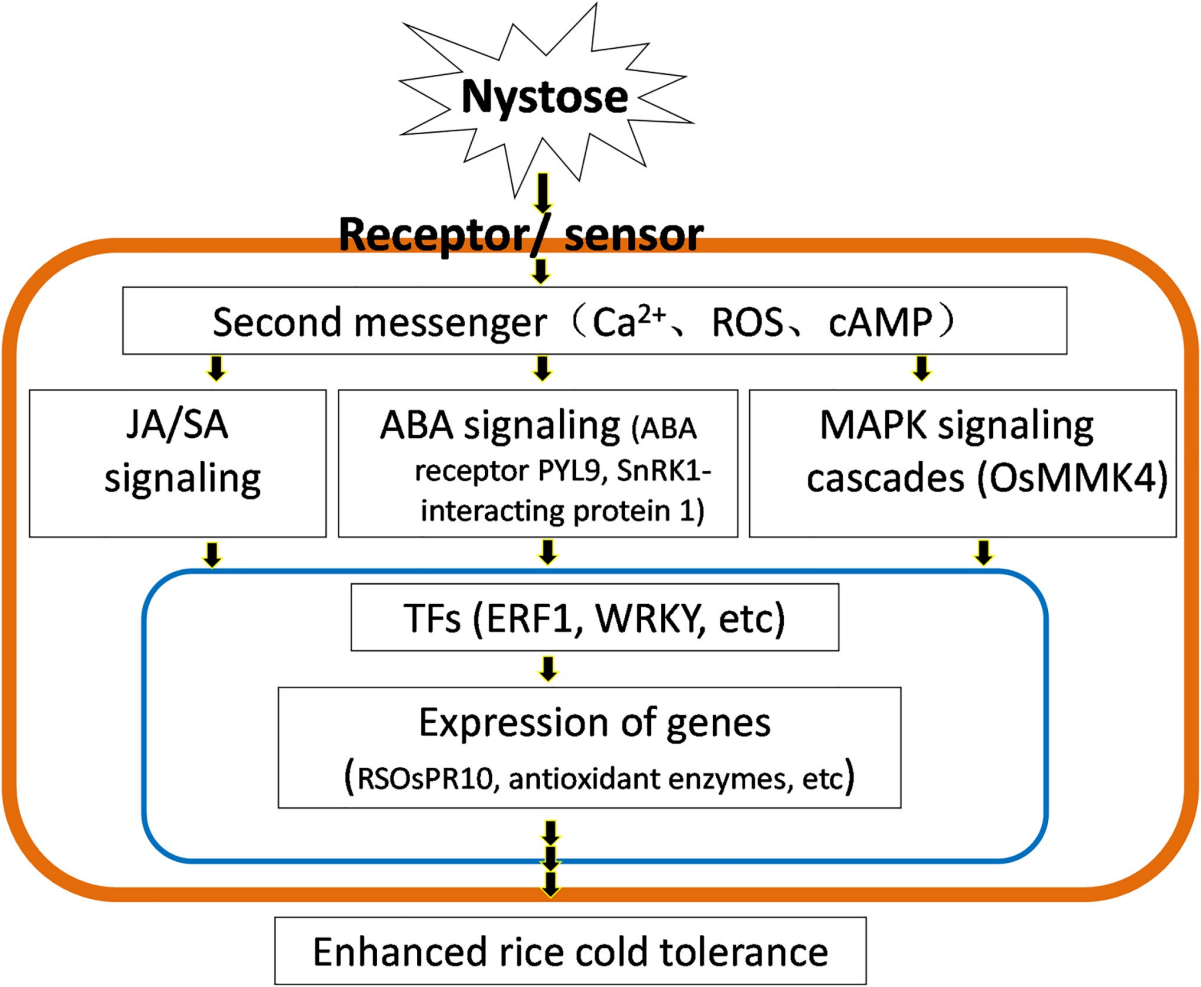
Flow chart of the mechanisms underlying the improvement in cold tolerance of primary rice roots by nystose.

Two upregulated DEPs involved in hormonal signaling, Q5Z8S0 (abscisic acid receptor PYL9) and Q10AI2 (SnRK1-interacting protein 1) were identified (S1 File). These proteins are likely functioning as components of ABA signaling pathways involved in stress responses. Interestingly, nystose-treated primary rice roots had a higher ABA level under cold stress than untreated roots (S9 Fig), indicating that nystose promotes ABA biosynthesis. Therefore, it can be concluded that nystose regulates the response of rice primary roots to cold stress by ABA-dependent signaling (Fig 5).

In addition to hormonal signaling, the MAPK cascade may be involved in mediating the effects of nystose. MAPK cascade comprises modules that transduce extracellular signals into a variety of cellular responses. Plant MAPK cascades have been implicated in the development and stress responses. We have identified an upregulated protein Q6K4Q0 that was confirmed as the mitogen-activated protein kinase 4 (OsMKK4). OsMKK4 is strongly regulated by cold and salt stress [54]. Thus, OsMKK4-dependent MAPK signaling cascades may be implicated in the nystose-induced improvement of cold tolerance by primary rice roots (Fig 5).

## Conclusions

This study integrates the results of morphologic, physiologic, and comparative proteomics analyses of primary rice roots to address the molecular mechanism by which nystose improves plant tolerance to cold stress. Nystose promotes the growth of primary rice roots, possibly by upregulating the expression of RSOsPR10. In turn, the changes in RSOsPR10 level associated with the response of rice roots to cold stress are probably regulated by the JA, SA, and ABA signaling pathways. Furthermore, OsMKK4-dependent MAPK signaling cascades may be involved in the nystose-induced tolerance of rice roots to the cold. Therefore, our findings indicate that nystose acts as an immunostimulator of the response to cold stress by multiple signaling pathways.

## Supporting information

S1 Fig Phenotypes of rice roots under the condition of normal growth. Phenotypes of rice primary roots from soaking seeds in water after growing at 25°C for 3 d (a), 5 d (c) and 7 d (e). Phenotypes of rice primary roots from soaking seeds in 75 mg/L nystose after growing at 25°C for 3 d (b), 5 d (d) and 7 d (f).
(TIF)

S2 Fig Protein expression profiles of rice roots treated with water and nystose under normal growth and cold stress conditions. Number of DEPs (P<0.05).
(TIF)

S3 Fig GO classification of the DEPs of rice roots treated with water and nystose grown under normal and cold stress conditions. (A) NW-vs-NN (24h), rice roots grown at 25°C for 24 h after water and nystose treatments. (B) CW-vs-CN (24h), rice roots grown at 4°C for 24 h after water and nystose treatments. (C) NW-vs-NN (48 h), rice roots grown at 25°C for 48 h after water and nystose treatments. (D) CW-vs-CN (48 h), rice roots grown at 4°C for 48 h after water and nystose treatments. (E) NW-vs-NN (recovery), rice roots grown at 25°C for 7 d after water and nystose treatments. (F) CW-vs-CN (recovery), rice roots grown at 4°C for 2 d and then at 25°C for 5 d after water and nystose treatments.
(TIF)

S4 Fig KEGG pathway enrichment analysis based on DEPs of control and nystose-treated rice roots grown under normal and cold stress conditions. (A) NW-vs-NN (24h), water- or nystose-treated rice roots grown at 25°C for 24 h. (B) CW-vs-CN (24h), water- or nystose-treated rice roots grown at 4°C for 24 h. (C) NW-vs-NN (48h), water- or nystose-treated rice roots grown at 25°C for 48 h. (D) CW-vs-CN (48h), water- or nystose-treated rice roots grown at 4°C for 48 h. (E) NW-vs-NN (recovery), water- or nystose-treated rice roots grown at 25°C for 7 d. (F) CW-vs-CN (recovery), water- or nystose-treated rice roots grown at 4°C for 2 d and then at 25°C for 5 d.
(TIF)

S5 Fig GO classification of up-regulated DEPs of rice roots treated with water and nystose under normal and cold stress conditions. (A) NW-vs-NN (24 h), rice roots that grew at 25°C for 24 h after treatment with water and nystose. (B) NW-vs-NN (48 h), rice roots that grew at 25°C for 48 h after treatment with water and nystose. (C) CW-vs-CN (48h), rice roots that grew at 4°C for 48 h after treatment with water and nystose. (D) NW-vs-NN (recovery), rice roots that grew at 25°C for 7 d after treatment with water and nystose. (E) CW-vs-CN (recovery), rice roots that grew at 4°C for 2 d and then at 25°C for 5 d after treatment with water and nystose.
(TIF)

S6 Fig GO classification of down-regulated DEPs in rice roots treated with water and nystose under normal and cold stress conditions. (A) NW-vs-NN (24 h), rice roots that grew at 25°C for 24 h after treatment with water and nystose. (B) CW-vs-CN (24 h), rice roots that grew at 4°C for 24 h after treatment with water and nystose. (C) NW-vs-NN (48 h), rice roots that grew at 25°C for 48 h after treatment with water and nystose. (D) CW-vs-CN (48 h), rice roots that grew at 4°C for 48 h after treatment with water and nystose. (E) NW-vs-NN (recovery), rice roots that grew at 25°C for 7 d after treatment with water and nystose. (F) CW-vs-CN (recovery), rice roots that grew at 4°C for 2 d and then at 25°C for 5 d after treatment with water and nystose.
(TIF)

S7 Fig Pathway enrichment analysis based on the upregulated DEPs of rice roots treated with water and nystose under the condition of normal growth and cold stress. (A) NW-vs-NN (24h), rice roots that grew at 25°C for 24 h after treatment with water and nystose. (B) NW-vs-NN (48h), rice roots that grew at 25°C for 48 h after treatment with water and nystose. (C) CW-vs-CN (48h), rice roots that grew at 4°C for 48 h after treatment with water and nystose.
(TIF)

S8 Fig Pathway enrichment analysis based on the downregulated DEPs of rice roots treated with water and nystose under normal and cold stress conditions. (A) NW-vs-NN (24h), rice roots that grew at 25°C for 24 h after treatment with water and nystose. (B) NW-vs-NN (48h), rice roots that grew at 25°C for 48 h after treatment with water and nystose. (C) CW-vs-CN (48h), rice roots that grew at 4°C for 48 h after treatment with water and nystose. (D) NW-vs-NN (recovery), rice roots that grew at 25°C for 7 d after treatment with water and nystose. (E) CW-vs-CN (recovery), rice roots that grew at 4°C for 2 d and then at 25°C for 5 d after treatment with water and nystose.
(TIF)

S9 Fig ABA content in rice primary roots grown at 25°C and 4°C for 48 h after treatment with water and nystose. HPLC-MS/MS was used for the analysis of ABA in rice roots. Experiments were repeated on at least three occasions. Data are the mean ± SE (n = 3). Different lower case letters on the top of each of the bars indicate significant differences (P<0.05, two-way ANOVA followed by the Tukey test).
(TIF)

S1 File DEPs following the analysis of Perseus software. NW-vs-NN (24 h): rice roots that grew at 25°C for 24 h after treatment with water and nystose. CW-vs-CN (24h): rice roots that grew at 4°C for 24 h after treatment with water and nystose. NW-vs-NN (48h): rice roots that grew at 25°C for 48 h after treatment with water and nystose. CW-vs-CN (48h): rice roots that grew at 4°C for 48 h after treatment with water and nystose. NW-vs-NN (recovery): rice roots that grew at 25°C for 7 d after treatment with water and nystose. CW-vs-CN (recovery): rice roots that grew at 4°C for 2 d and then at 25°C for 5 d after treatment with water and nystose.
(XLSX)

S2 File List of upregulated proteins (P<0.05, fold change>1.5). NW-vs-NN (24h): rice roots that grew at 25°C for 24 h after treatment with water and nystose. CW-vs-CN (24h): rice roots that grew at 4°C for 24 h after treatment with water and nystose. NW-vs-NN (48h): rice roots that grew at 25°C for 48 h after treatment with water and nystose. CW-vs-CN (48h): rice roots that grew at 4°C for 48 h after treatment with water and nystose. NW-vs-NN (recovery): rice roots that grew at 25°C for 7 d after treatment with water and nystose. CW-vs-CN (recovery): rice roots that grew at 4°C for 2 d and then at 25°C for 5 d after treatment with water and nystose.
(XLSX)

S3 File Lists of down-regulated proteins (P<0.05, fold change<2/3). NW-vs-NN (24h): rice roots that grew at 25°C for 24 h after treatment with water and nystose. CW-vs-CN (24h): rice roots that grew at 4°C for 24 h after treatment with water and nystose. NW-vs-NN (48h): rice roots that grew at 25°C for 48 h after treatment with water and nystose. CW-vs-CN (48h): rice roots that grew at 4°C for 48 h after treatment with water and nystose. NW-vs-NN (recovery): rice roots that grew at 25°C for 7 d after treatment with water and nystose. CW-vs-CN (recovery): rice roots that grew at 4°C for 2 d and then at 25°C for 5 d after treatment with water and nystose.
(XLSX)

S4 File Matched protein datasets following the analysis of MaxQuant software. NW-NN-CW-CN (24 h): rice roots that grew at 25°C and 4°C for 24 h after treatment with water and nystose. NW-NN-CW-CN (48 h): rice roots that grew at 25°C and 4°C for 48 h after treatment with water and nystose. NW-NN-CW-CN (recovery): rice roots that grew at 25°C for 7 d and 4°C for 2 d and then at 25°C for 5 d after treatment with water and nystose.
(XLSX)

S5 File List of peptides for antibody preparation. (XLSX)
