# Supplementary figures and images for: Nystose regulates the response of rice roots to cold stress via multiple signaling pathways: A comparative proteomics analysis
Source: PLoS One. 2020 Sep 3;15(9):e0238381. doi: 10.1371/journal.pone.0238381 (PMC7470417; doi:10.1371/journal.pone.0238381)

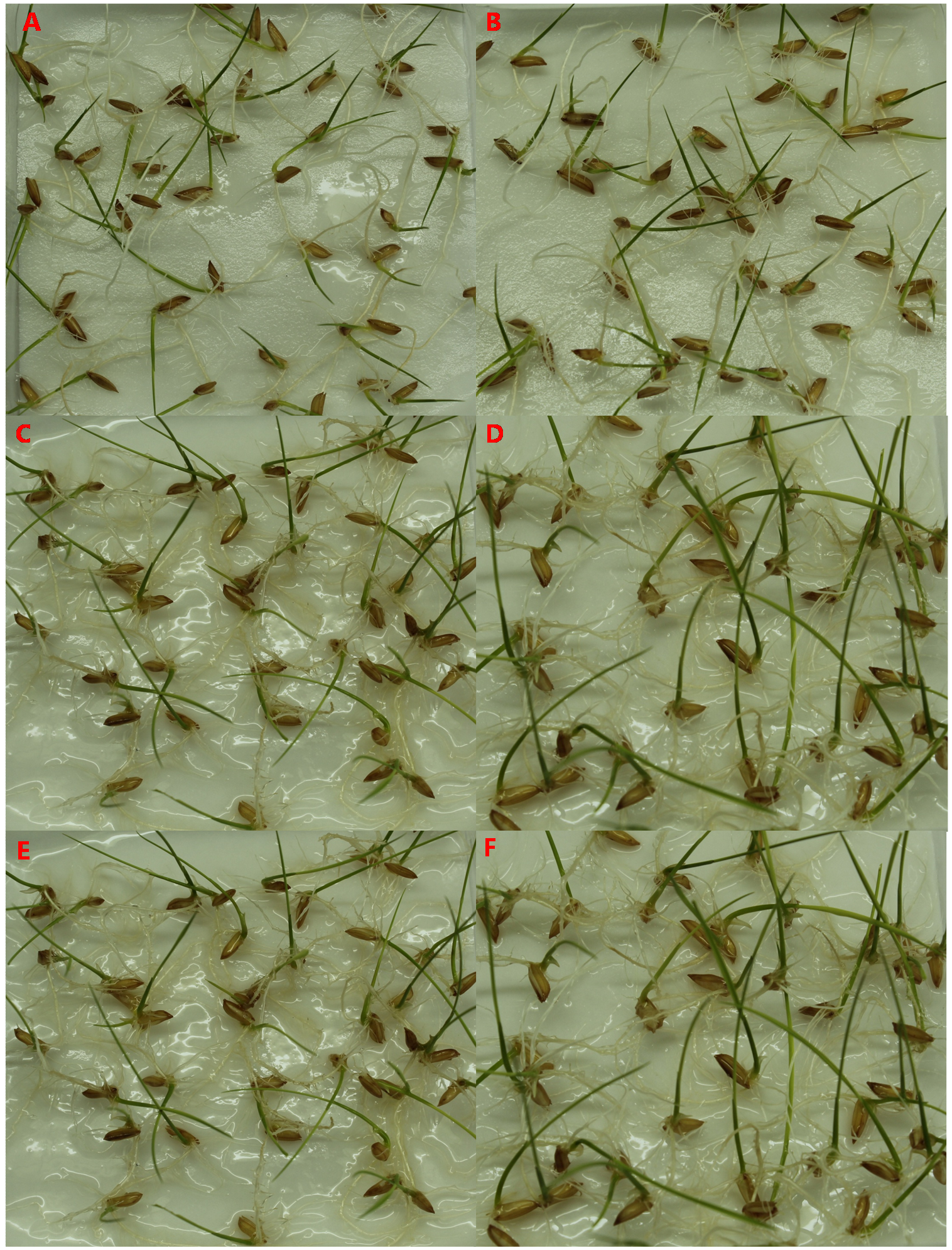

Supplement: S1 Fig — Phenotypes of rice primary roots from soaking seeds in water after growing at 25°C for 3 d (a), 5 d (c) and 7 d (e). Phenotypes of rice primary roots from soaking seeds in 75 mg/L nystose after growing at 25°C for 3 d (b), 5 d (d) and 7 d (f). (TIF) [file pone.0238381.s001.tif]

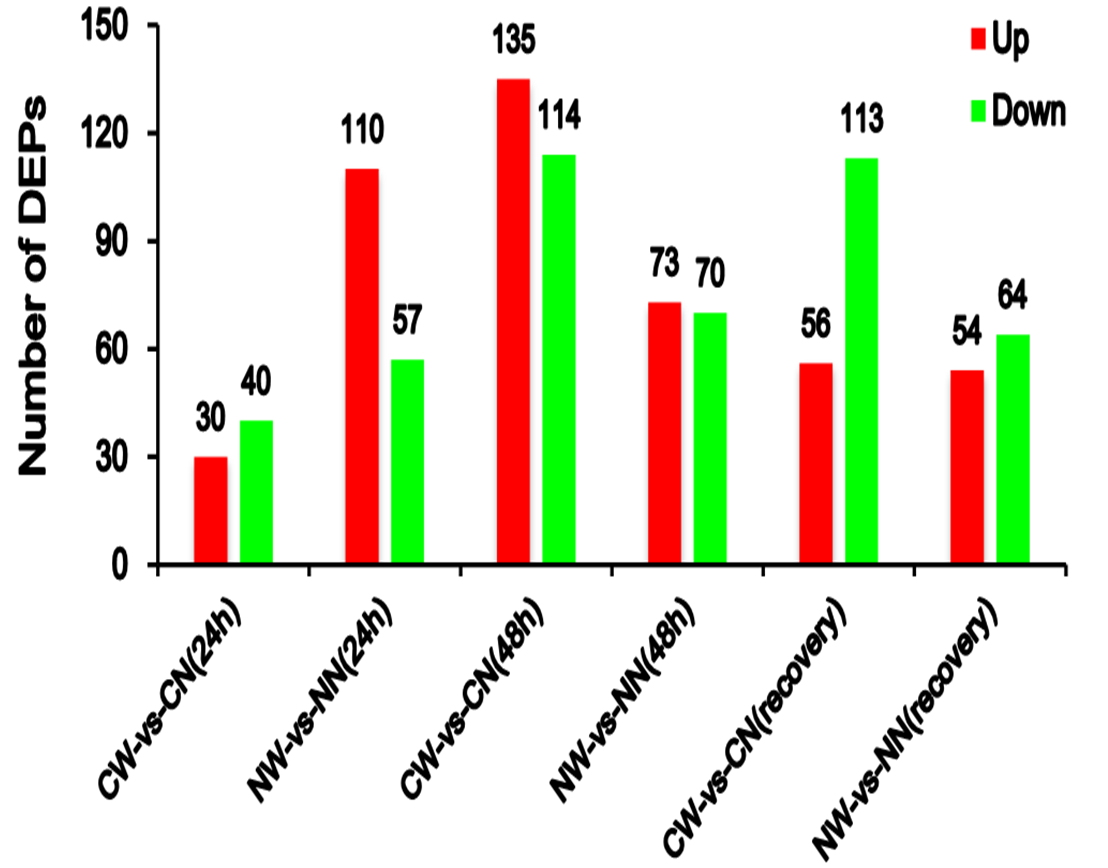

Supplement: S2 Fig — Number of DEPs (P<0.05). (TIF) [file pone.0238381.s002.tif]

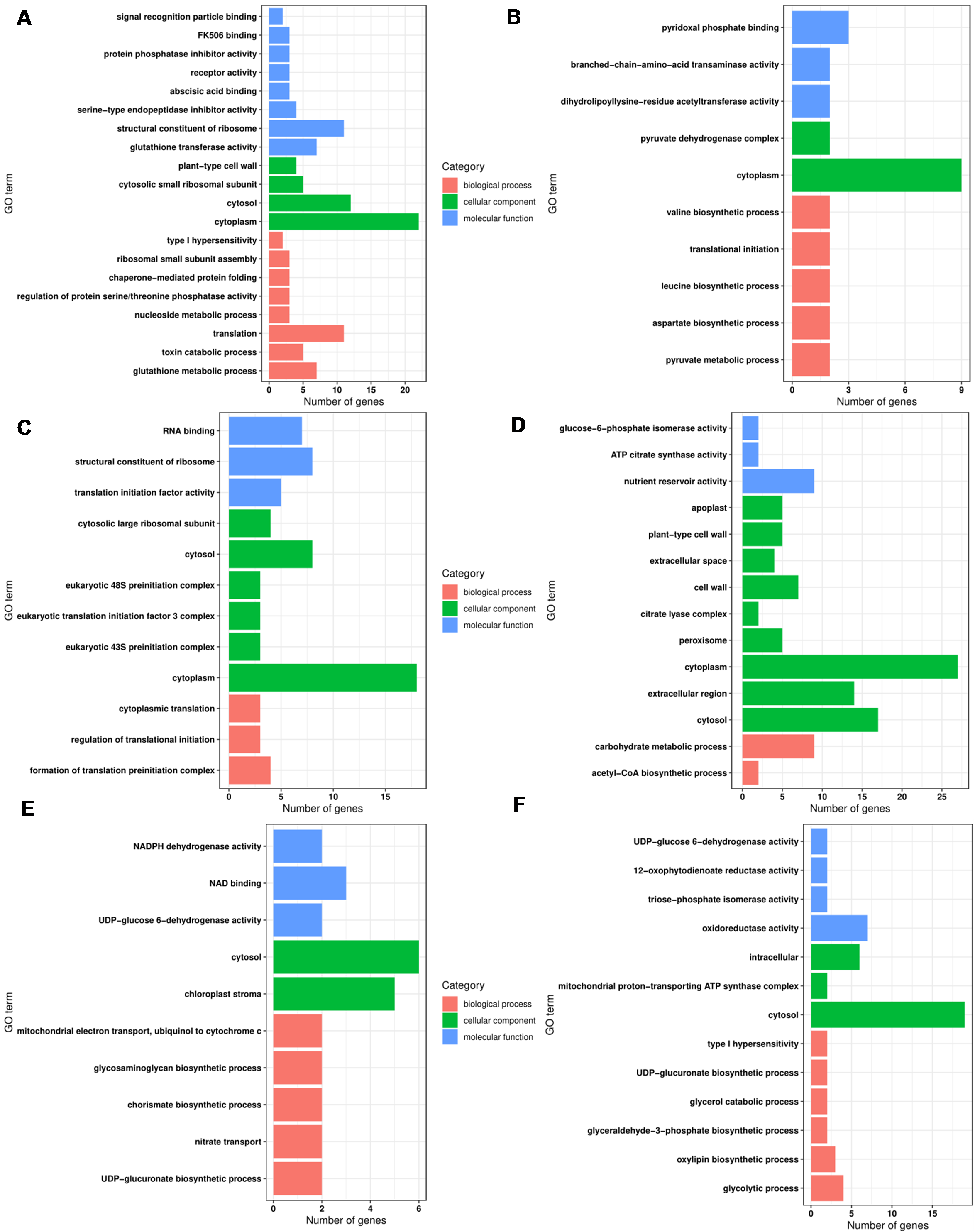

Supplement: S3 Fig — (A) NW-vs-NN (24h), rice roots grown at 25°C for 24 h after water and nystose treatments. (B) CW-vs-CN (24h), rice roots grown at 4°C for 24 h after water and nystose treatments. (C) NW-vs-NN (48 h), rice roots grown at 25°C for 48 h after water and nystose treatments. (D) CW-vs-CN (48 h), rice roots grown at 4°C for 48 h after water and nystose treatments. (E) NW-vs-NN (recovery), rice roots grown at 25°C for 7 d after water and nystose treatments. (F) CW-vs-CN (recovery), rice roots grown at 4°C for 2 d and then at 25°C for 5 d after water and nystose treatments. (TIF) [file pone.0238381.s003.tif]

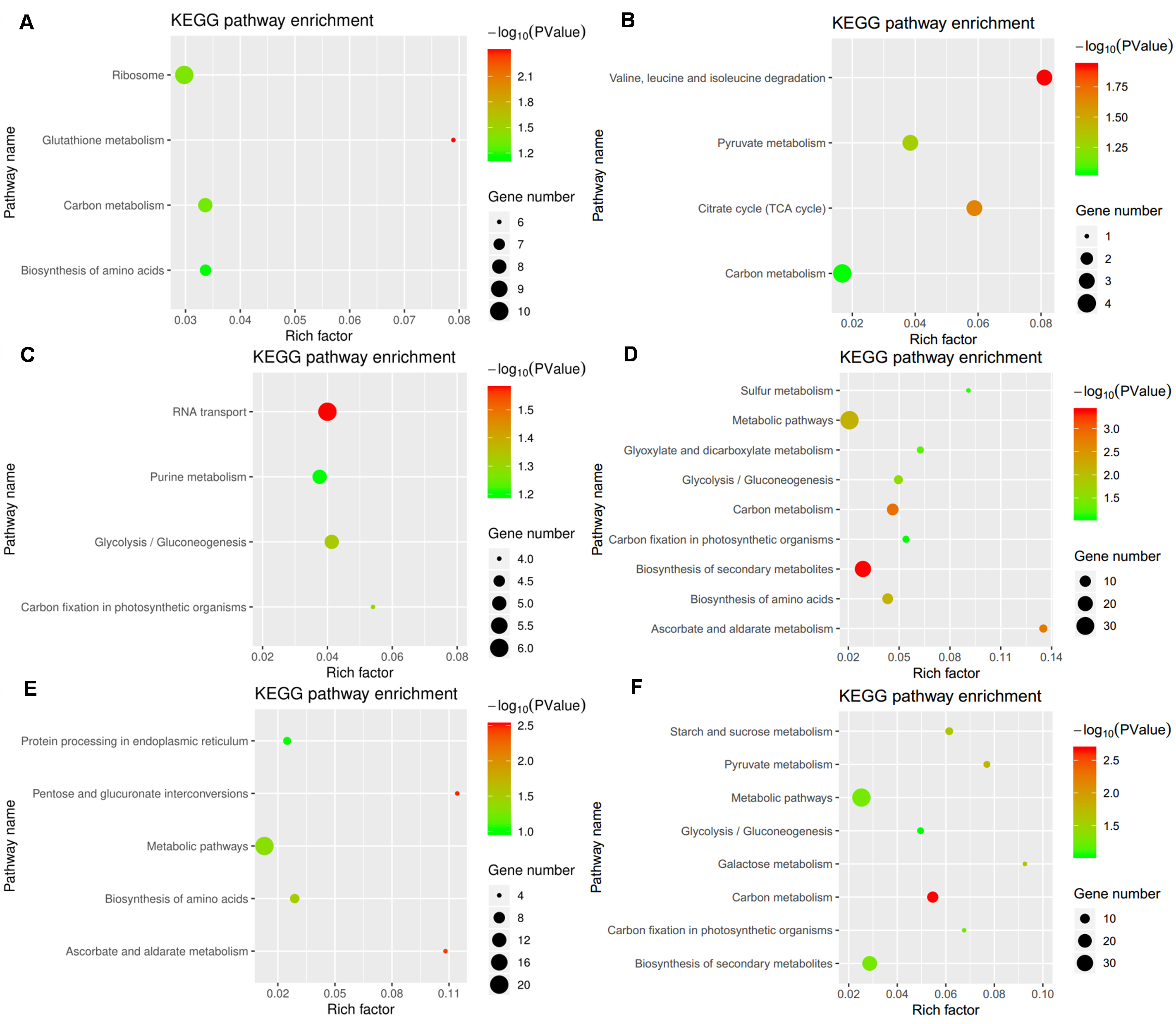

Supplement: S4 Fig — (A) NW-vs-NN (24h), water- or nystose-treated rice roots grown at 25°C for 24 h. (B) CW-vs-CN (24h), water- or nystose-treated rice roots grown at 4°C for 24 h. (C) NW-vs-NN (48h), water- or nystose-treated rice roots grown at 25°C for 48 h. (D) CW-vs-CN (48h), water- or nystose-treated rice roots grown at 4°C for 48 h. (E) NW-vs-NN (recovery), water- or nystose-treated rice roots grown at 25°C for 7 d. (F) CW-vs-CN (recovery), water- or nystose-treated rice roots grown at 4°C for 2 d and then at 25°C for 5 d. (TIF) [file pone.0238381.s004.tif]

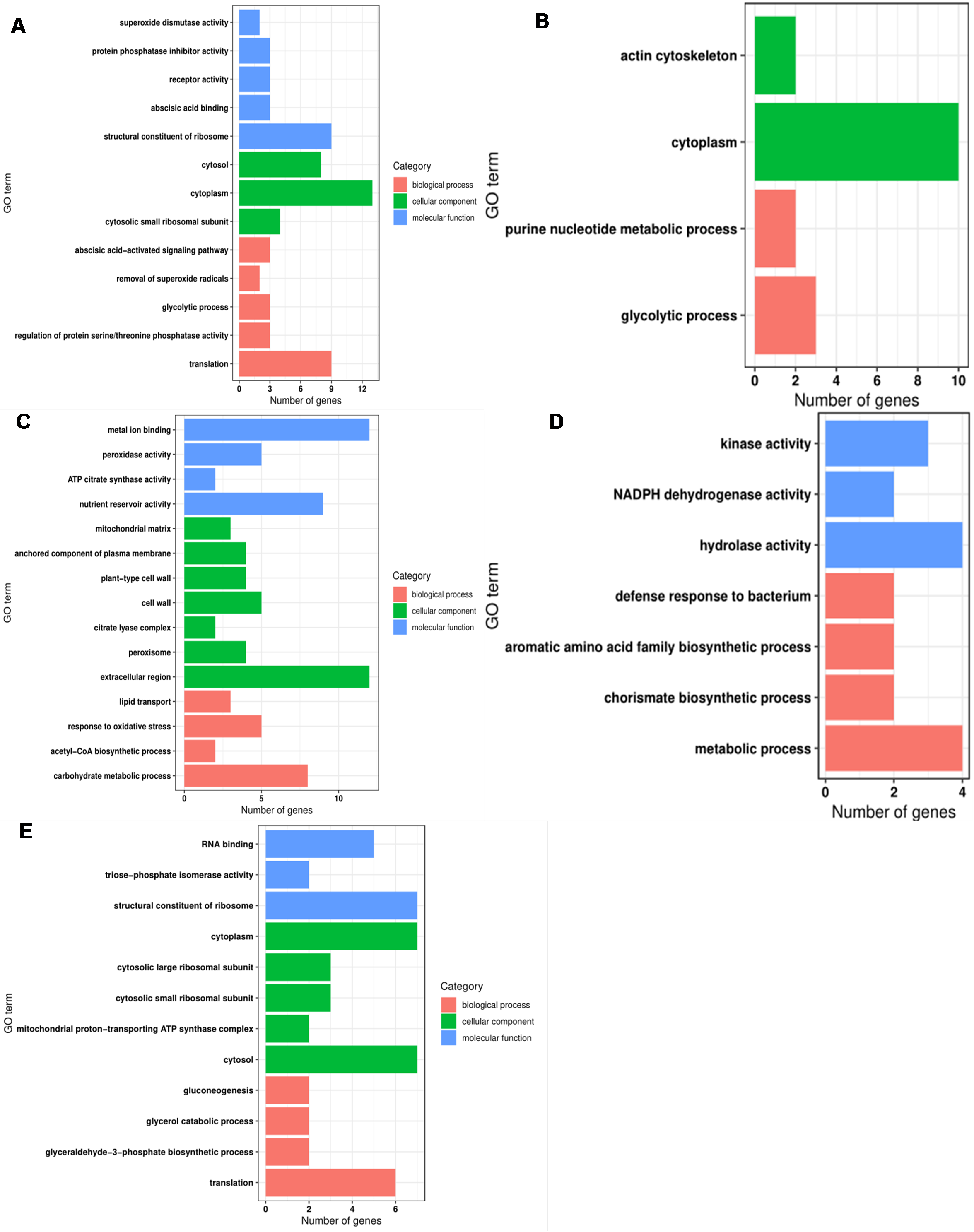

Supplement: S5 Fig — (A) NW-vs-NN (24 h), rice roots that grew at 25°C for 24 h after treatment with water and nystose. (B) NW-vs-NN (48 h), rice roots that grew at 25°C for 48 h after treatment with water and nystose. (C) CW-vs-CN (48h), rice roots that grew at 4°C for 48 h after treatment with water and nystose. (D) NW-vs-NN (recovery), rice roots that grew at 25°C for 7 d after treatment with water and nystose. (E) CW-vs-CN (recovery), rice roots that grew at 4°C for 2 d and then at 25°C for 5 d after treatment with water and nystose. (TIF) [file pone.0238381.s005.tif]

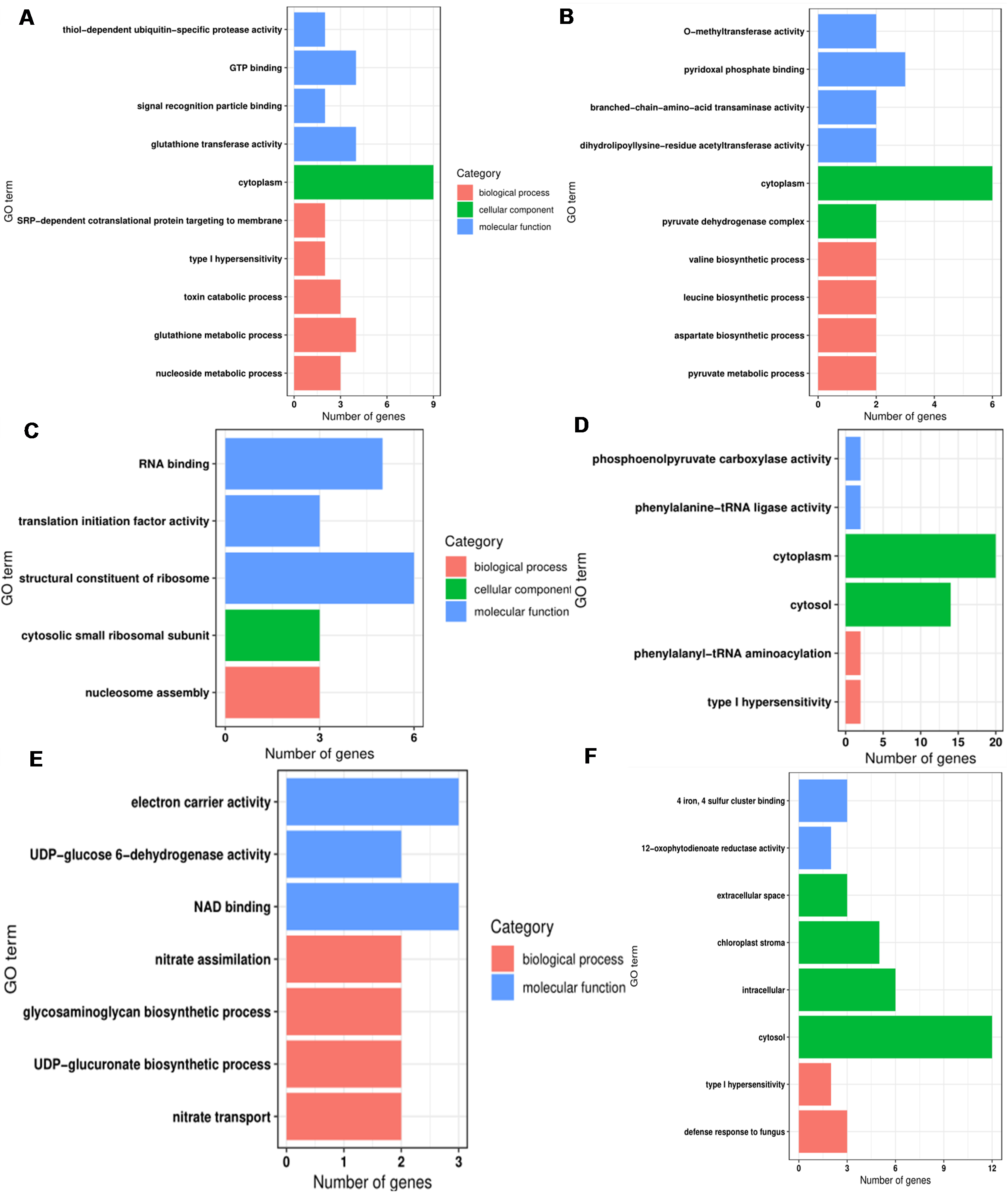

Supplement: S6 Fig — (A) NW-vs-NN (24 h), rice roots that grew at 25°C for 24 h after treatment with water and nystose. (B) CW-vs-CN (24 h), rice roots that grew at 4°C for 24 h after treatment with water and nystose. (C) NW-vs-NN (48 h), rice roots that grew at 25°C for 48 h after treatment with water and nystose. (D) CW-vs-CN (48 h), rice roots that grew at 4°C for 48 h after treatment with water and nystose. (E) NW-vs-NN (recovery), rice roots that grew at 25°C for 7 d after treatment with water and nystose. (F) CW-vs-CN (recovery), rice roots that grew at 4°C for 2 d and then at 25°C for 5 d after treatment with water and nystose. (TIF) [file pone.0238381.s006.tif]

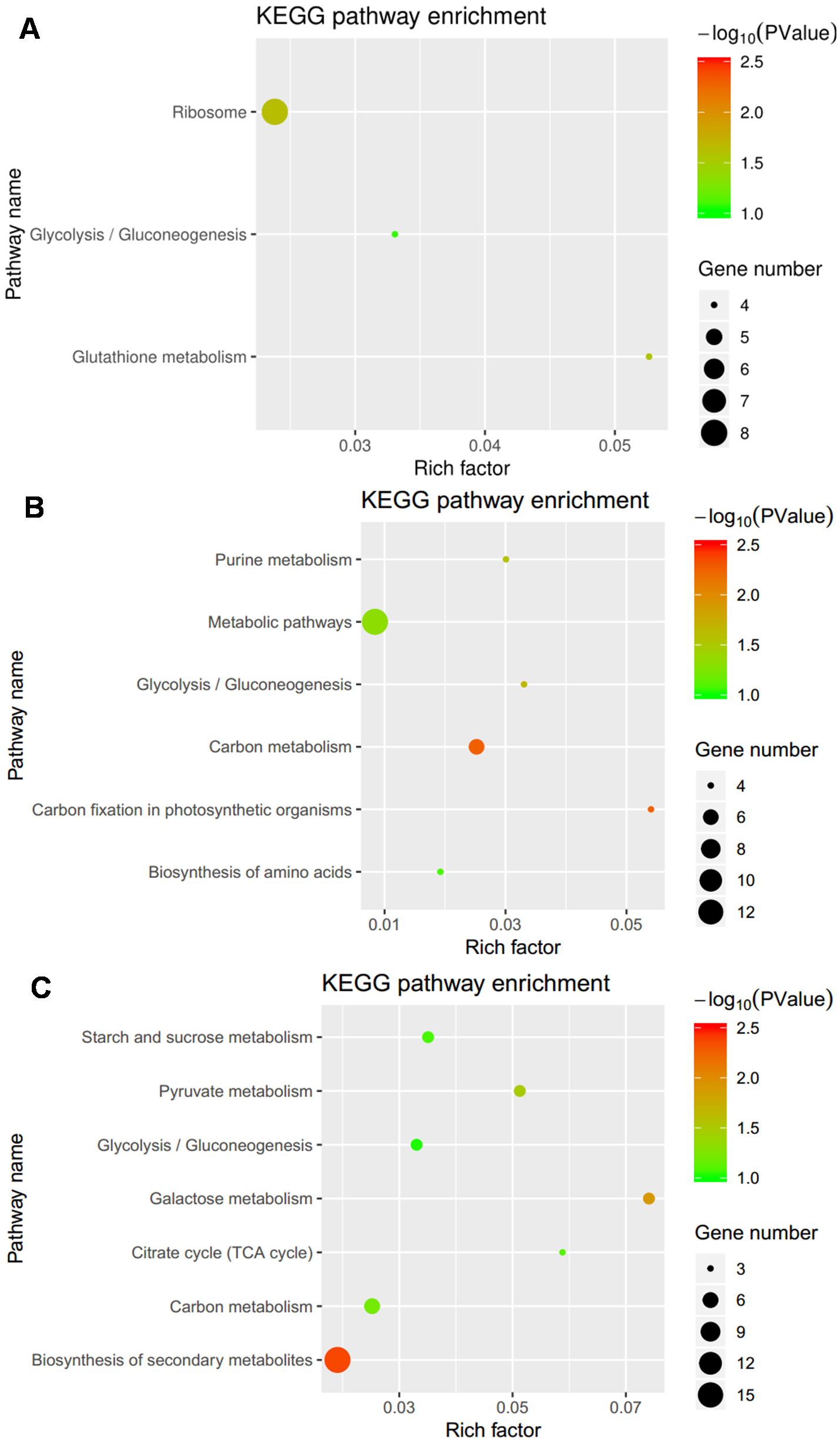

Supplement: S7 Fig — (A) NW-vs-NN (24h), rice roots that grew at 25°C for 24 h after treatment with water and nystose. (B) NW-vs-NN (48h), rice roots that grew at 25°C for 48 h after treatment with water and nystose. (C) CW-vs-CN (48h), rice roots that grew at 4°C for 48 h after treatment with water and nystose. (TIF) [file pone.0238381.s007.tif]

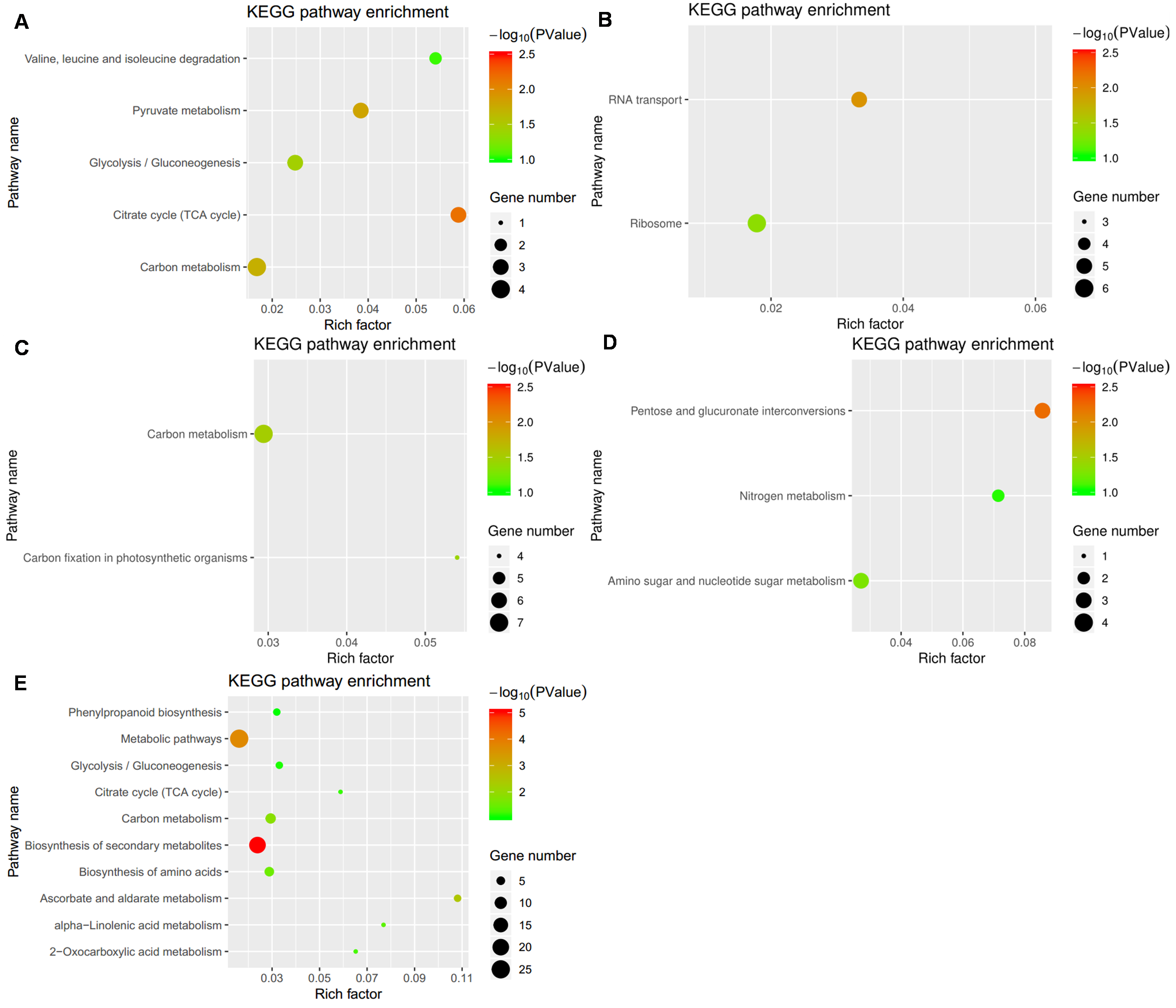

Supplement: S8 Fig — (A) NW-vs-NN (24h), rice roots that grew at 25°C for 24 h after treatment with water and nystose. (B) NW-vs-NN (48h), rice roots that grew at 25°C for 48 h after treatment with water and nystose. (C) CW-vs-CN (48h), rice roots that grew at 4°C for 48 h after treatment with water and nystose. (D) NW-vs-NN (recovery), rice roots that grew at 25°C for 7 d after treatment with water and nystose. (E) CW-vs-CN (recovery), rice roots that grew at 4°C for 2 d and then at 25°C for 5 d after treatment with water and nystose. (TIF) [file pone.0238381.s008.tif]

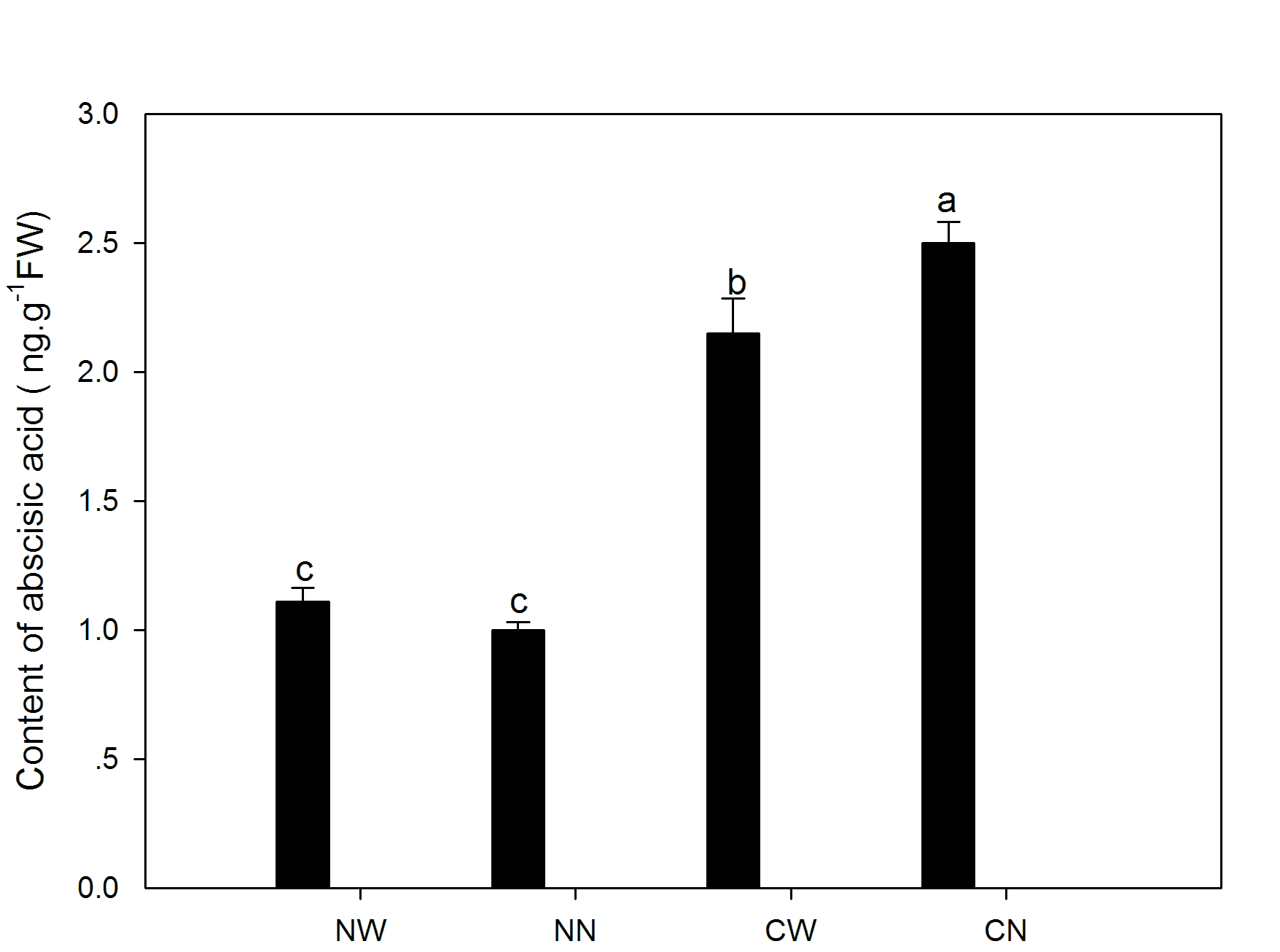

Supplement: S9 Fig — HPLC-MS/MS was used for the analysis of ABA in rice roots. Experiments were repeated on at least three occasions. Data are the mean ± SE (n = 3). Different lower case letters on the top of each of the bars indicate significant differences (P<0.05, two-way ANOVA followed by the Tukey test). (TIF) [file pone.0238381.s009.tif]
